# Consensus on the diagnosis and treatment of adult necrotizing fasciitis (2025 edition)

**DOI:** 10.1093/burnst/tkaf031

**Published:** 2025-06-12

**Authors:** Ling Zhou, Haisheng Li, Gaoxing Luo, the CANF experts group representing the Chinese Burn Association, Editorial Board of the Chinese Journal of Burns and Wounds, Burn Medicine Branch of China International Exchange and Promotion Association for Medical and Healthcare

**Affiliations:** Institute of Burn Research, The First Affiliated Hospital of Army Medical University, (the Third Military Medical University) Gaotanyan Street, Chongqing 400038, China; Institute of Burn Research, The First Affiliated Hospital of Army Medical University, (the Third Military Medical University) Gaotanyan Street, Chongqing 400038, China; Institute of Burn Research, The First Affiliated Hospital of Army Medical University, (the Third Military Medical University) Gaotanyan Street, Chongqing 400038, China

**Keywords:** Necrotizing fasciitis, Multidisciplinary, Consensus

## Abstract

Necrotizing fasciitis (NF) is a disease characterized by severe infection of the skin and its underlying soft tissues as the initial symptom. NF is known for its difficulty in early diagnosis and rapid progression. If not treated promptly, NF can quickly evolve into systemic infection, sepsis, and multiple organ failure, and it may even lead to patient death. Currently, many controversies and challenges remain in clinical practice for the diagnosis and treatment of NF. To promote the standardization of NF diagnosis and treatment, Chinese Burn Association, *Editorial Board of the Chinese Journal of Burns and Wounds*, and Burn Medicine Branch of China International Exchange and Promotion Association for Medical and Health Care, based on the latest relevant guidelines, literature, and clinical practice experience and in accordance with the principles of evidence-based medicine, have jointly developed the Consensus on the Diagnosis and Treatment of Adult Necrotizing Fasciitis (2025 Edition) through repeated discussion and voting. This consensus aims to provide scientific and standardized guidance for clinical diagnosis and treatment.

Highlights

- Currently, no standardized protocol exists for diagnosing and treating necrotizing fasciitis. This consensus, formulated through multidisciplinary expert discussions, integrates recent guidelines, literature, and clinical practice experiences, adhering to evidence-based medicine principles.
- This document presents recommendations for standardizing the diagnosis and treatment of necrotizing fasciitis. These findings serve as a basis for further refinement of diagnostic and therapeutic strategies for this condition.

## Background

Necrotizing fasciitis (NF) is a rare yet highly aggressive soft tissue infection distinguished by rapid necrosis of the fascia and subcutaneous tissues. NF is often accompanied by systemic septic shock and multiple organ dysfunction syndrome, posing a significant threat to patient survival. The disease is characterized by its severe nature and rapid progression, with an incidence rate ranging from 0.3 to 15 cases per 100 000 individuals and a mortality rate as high as 25%–35% [1]. The insidious onset and lack of distinctive early symptoms contribute to a misdiagnosis rate of 50%–75% [2]. Despite the remarkable advancements in anti-infective therapy, intensive care, and surgical techniques, NF remains a formidable challenge in the realms of clinical emergency and critical care. In recent years, the global incidence of NF has been increasing, which can be attributed to factors such as population aging, the increasing prevalence of chronic diseases, and the widespread use of broad-spectrum antibiotics. A multitude of risk factors are associated with NF, including diabetes mellitus (DM), chronic liver disease, chronic kidney disease, autoimmune diseases, long-term use of immunosuppressive drugs (e.g. prednisolone), malignancies, HIV infection, malnutrition, obesity, intravenous drug use, major surgeries, trauma, and burns [3–5]. The spectrum of pathogens involved in NF has become increasingly intricate. In addition to common pathogens such as group A β-hemolytic streptococcus (GAS) and mixed anaerobic infections, the detection rate of drug-resistant strains (e.g. methicillin-resistant *Staphylococcus aureus*) and rare pathogens (e.g. *Vibrio vulnificus*) has increased, further complicating clinical diagnosis and treatment. Currently, there is a lack of consensus or unified guidelines for the diagnosis and treatment of NF in China, and numerous controversies and challenges persist in clinical practice. This consensus aims to address these issues by systematically reviewing the etiological characteristics, diagnostic criteria, graded treatment strategies, and rehabilitation management strategies for NF on the basis of the latest domestic and international medical evidence in conjunction with the characteristics of clinical practice in China. Through collaboration among multidisciplinary experts, this consensus seeks to enhance early recognition, optimize treatment protocols, reduce mortality rates and disability rates, and ultimately improve patient quality of life.

## Methods

### Registration and planning

This consensus was registered in both Chinese and English on the International Platform for Guideline Registration and Transparency (http://www.guidelines-registry.cn) before its development (registration number: PREPARE-2024CN393), and a detailed expert consensus plan was uploaded.

### Development team and process

The consensus was developed by a multidisciplinary team of experts, covering fields such as burn surgery, plastic surgery, orthopedics, general surgery, urology, emergency trauma, and evidence-based medicine. The working group included a chief expert responsible for overall implementation, an expert panel for quality assessment of evidence and formation of recommendations, a literature review team for literature search and evidence collection, an evidence evaluation team for preliminary assessment of evidence, and a drafting team for writing the consensus. The development was oriented toward clinical application, integrating domestic and international experiences in the diagnosis and treatment of NF and referencing the relevant literature. After one round of clinical question solicitation, two rounds of Delphi expert consultation, and two rounds of panel discussions, the final recommendations were formed.

### Clinical questions and literature search

The academic working group initially conducted a survey on the current treatment status of NF and preliminarily defined the scope of clinical questions through a literature search and analysis. Subsequently, expert group discussions and online questionnaire surveys were organized to ascertain the importance of the clinical questions. The clinical questions were structured according to the PICO principle (P: Population/Patient, I: Intervention, C: Comparison/Control, O: Outcome). For the literature search, the keywords included “necrotizing fasciitis,” “necrotising fasciitis,” “necrotizing soft tissue infection,” “Fournier's gangrene,” “Fournier's ulcer,” “hospital-acquired gangrene,” “acute infectious gangrene,” “purulent fasciitis,” “streptococcal gangrene,” etc. The search covered diagnostic and therapeutic advances in NF. The databases searched included clinical decision support systems, domestic and international clinical practice guidelines, and literature databases, with a search period from the inception of these databases up to 31 January 2025. The study subjects included all NF-related studies from various sites. The included literature types were guidelines, clinical decisions, evidence summaries, recommended practices, expert consensuses, meta-analyses, systematic reviews, randomized controlled trials, prospective cohort studies, retrospective case-control studies, clinical case reports, and case series, while study protocols, conference presentations, abstracts, incomplete information, inaccessible full texts, and duplicate publications were excluded.

### Evidence grading and recommendation formulation

The drafting team utilized the Grading of Recommendations Assessment, Development, and Evaluation (GRADE) system to grade the evidence-based findings, categorizing the quality of evidence into four levels—high, moderate, low, and very low (Table 1) [6]. After the expert panel adjusted and modified the recommendations, the panel scored them on the basis of the level of agreement, which was divided into five dimensions as follows: “strongly agree,” “agree,” “neutral,” “disagree,” and “strongly disagree.” The ratio of “strongly agree” to “agree” was used as the reference for the strength of the recommendation. The formulation of recommendation levels considered factors such as the quality of evidence, patient values, patient preferences, cost-effectiveness, and feasibility of interventions. The recommendation levels were divided as follows: (i) strong recommendation, where on the basis of existing evidence-based medical evidence or common consensus the measure is considered beneficial or effective for the vast majority of patients (“strongly agree” + “agree” ratio ≥ 90%), and (ii) weak recommendation, where the clinical benefit of the measure is not sufficiently supported by evidence or the benefits and risks are balanced, and its use should be carefully considered on the basis of individual patient circumstances (60% ≤ “strongly agree” + “agree” ratio < 90%). A “strongly agree” + “agree” ratio < 60% was not included. The detailed criteria of evidence levels and recommendation tiers are presented in Table 1.

**Table 1:** Descriptions of the strength of the recommendations and their quality of evidence based on the GRADE system

Item	Definition
Recommendation strength	
Strong	Strongly recommend adoption
Weak	Recommend adoption with clinical prudence
Quality of evidence	
High	Evidence from well-conducted RCTs^a^ in populations with trauma or necrotizing fasciitis, or evidence from systematic reviews/meta-analyses and authoritative guidelines that include at least one high-quality RCT
Moderate	Evidence from RCTs with lower quality, or evidence from well-conducted nonrandomized studies, cohort studies, case-control studies, multicenter registries, or high-quality RCTs in populations not specifically identified with trauma or necrotizing fasciitis
Low	Evidence from observational studies, or evidence from nonrandomized studies, cohort studies, or case-control studies in populations not specifically identified with trauma or necrotizing fasciitis
Very low	Evidence from case reports, clinical summaries, or observational studies in populations not specifically identified with trauma or necrotizing fasciitis

^a^RCT, randomized controlled trial

### Consensus formation

Finally, the academic working group compiled the expert consultation results and voting results on the consistency of the recommendations to formulate this consensus.

## Clinical questions and recommendations

### Clinical question 1: overview of necrotizing fasciitis

Recommendation 1 (highly recommended): NF is a rare yet extremely severe purulent soft tissue infection. NF primarily spreads rapidly along the fascial plane and typically involves the subcutaneous tissue, superficial fascia, and deep fascia, while sparing the muscular layer. NF has an acute onset and progresses rapidly, usually accompanied by systemic symptoms of infection and toxicity. NF is characterized by a low incidence but high mortality rate. Individuals with compromised immune function are particularly susceptible to this type of infection (evidence level: high).

Rationale: Necrotizing fasciitis is a severe purulent soft tissue infection caused by a mixture of various bacteria, and it is characterized by rapid necrosis of the skin, subcutaneous tissue, and fascia. Once pathogens invade the skin and subcutaneous tissue, they can rapidly spread along the deep and superficial fascia, as well as the potential spaces between tendons. This spread often leads to thrombus formation within blood vessels, resulting in ischemia and subsequent necrosis of the affected skin, subcutaneous tissue, and fascia. However, the deep muscle layers are typically spared. In severe cases, NF can progress to systemic infection, causing sepsis and multiple organ dysfunction, and it may even be life-threatening.

Upon invading subcutaneous tissue, pathogenic bacteria release various toxins and enzymes during the infection process. These substances disrupt the local tissue structure and function, leading to acute and progressive necrosis. Simultaneously, activated immune cells release many inflammatory factors and cytokines, which further exacerbate the inflammatory response and tissue damage, resulting in widespread inflammation, congestion, and edema in tissues. Additionally, damage to blood vessels and lymphatics impairs local blood circulation and lymphatic drainage, preventing the timely clearance and repair of necrotic tissue. The fascial tissue, characterized by its loose structure, natural cavities, and relatively poor blood supply [7], facilitates covert spread of the infection along the fascia, thereby worsening the condition.

Extensive necrosis of the skin and subcutaneous tissue is a hallmark feature of NF. Because lymphatic pathways are often rapidly destroyed, lymphangitis and lymphadenitis are uncommon. However, adipose tissue frequently exhibits necrosis, liquefaction, and degeneration. Fascial tissue experiences extensive necrosis with the formation of undermining cavities that spread to surrounding tissues, which is one of the key characteristics of NF. Under microscopic examination, heavy infiltration of neutrophils in the fascia and fibrinoid necrosis of the walls of arteries and veins can be observed. The small vascular network in the skin and subcutaneous tissue undergoes inflammatory thrombosis, leading to tissue malnutrition and ischemic necrosis [8, 9].

NF frequently results from mixed infections involving both aerobic and anaerobic bacteria. Common pathogens include gram-positive bacteria (such as *Streptococcus pyogenes* and *S. aureus*), gram-negative bacteria (such as *Escherichia coli* and *Klebsiella pneumoniae*), and anaerobic bacteria (such as *Clostridium perfringens* and *Bacteroides fragilis*). In addition, infections caused by *V. vulnificus* and fungi are relatively rare [10–12].

### Clinical question 2: diagnosis of necrotizing fasciitis

Recommendation 2 (highly recommended): Early diagnosis is crucial for reducing the mortality rate of NF. A comprehensive evaluation of the patient’s susceptibility factors, medical history, characteristic clinical manifestations, and finger test results is recommended. In addition, diagnostic tools, such as the Laboratory Risk Indicator for Necrotizing Fasciitis (LRINEC) score, Site other than the lower limb, Immunosuppression, Age, Renal impairment, and Inflammatory markers (SIARI) score, and Laboratory and Anamnestic Risk Indicator for Necrotizing Fasciitis (LARINF) score, should be used in combination to assist in early risk identification and diagnosis (evidence level: high).

Rationale: NF typically has an insidious onset but progresses rapidly. Delayed diagnosis is a key factor contributing to its high mortality rate, thereby emphasizing the critical importance of early diagnosis. Diagnosis relies primarily on clinical manifestations, with necessary ancillary examinations serving as important adjuncts. Various scoring systems can provide additional support but should not be used in isolation.

Characteristic clinical manifestations: In the early stage, the skin presents mild localized erythema and swelling, which gradually progresses to erythematous plaques, edema, warmth, and tenderness. On palpation, the skin feels relatively soft and is often accompanied by severe pain that is disproportionate to the extent of visible symptoms. In the middle stage, the skin develops fissures, along with the formation of vesicles and bullae, as well as a certain degree of skin necrosis. In the late stage, hemorrhagic bullae, crepitus, and necrosis of the subcutaneous tissue manifest. The exudate is foul-smelling and purulent. The subcutaneous fat and fascia become edematous, with a thick, turbid, and blackish exudate that eventually liquefies and necroses. The exudate contains bacteria, necrotic tissue, and bacterial metabolic products. Owing to bacterial decomposition, hydrogen sulfide and other foul-smelling gases are produced, giving the exudate a distinct malodor. Common pathogens of NF include *E. coli*, *K. pneumoniae*, *S. aureus*, and *S. pyogenes*. These bacteria release multiple enzymes and toxins during infection, further exacerbating tissue necrosis and the foul smell of the exudate. The degree of odor can serve as a reference indicator for determining the severity of infection and the effectiveness of treatment. Additionally, owing to thrombosis in small vessels and damage to superficial subcutaneous nerves, the affected area may experience numbness, with pain paradoxically decreasing. Patients may also present with various systemic symptoms, such as fever, tachycardia, sepsis, electrolyte disturbances, and altered mental status, which may progress to septic shock and multiple organ failure. Even when intravenous antibiotics are administered, if the borders of erythema and skin induration continue to expand rapidly, this indicates ongoing disease progression and serves as an important early warning sign [13–15].

Finger test: After local anesthesia is administered to the suspected lesion site, an incision (with a suggested length of 2 cm) is made at the area of highest tension or most prominent fluctuation, extending down to the deep fascia. The tissue in the finger is then examined. The test result is positive and NF is highly suspected if there is no bleeding during exploration and the following characteristics are observed: foul-smelling discharge, poor muscle contractility, easy blunt dissection of subcutaneous tissue from the fascial layer using the finger, and a large amount of thin, “serous bloody fluid” (similar to “meat wash water” pus), which results from the lysis of neutrophils due to lecithinase and other bacterial toxins [16].

LRINEC score: In 2004, Wong *et al.* [17] first introduced the Laboratory Risk Indicator for Necrotizing Fasciitis score as an auxiliary tool for the early diagnosis of NF (Tables 2 and 3). Studies have demonstrated that when the LRINEC score is ≥6, it has a positive predictive value of 92% and a negative predictive value of 96% for diagnosing NF. The LRINEC score is not only valuable for early diagnosis but also closely correlates with patient mortality and amputation rates, making it a useful tool for risk assessment and prognostic prediction in NF patients [18–21]. However, the LRINEC score has certain limitations. The sensitivity of the LRINEC score may be influenced by certain factors, such as the site of infection, type of bacteria, renal function, and peripheral vascular disease, which can lead to missed diagnoses in some patients [22]. Importantly, while a high LRINEC score is indicative of NF, a low score cannot completely rule out the possibility of NF [20, 23, 24]. Therefore, in clinical practice, the LRINEC score should be used in conjunction with clinical manifestations and other diagnostic findings to increase the accuracy of NF diagnosis.

**Table 2:** LRINEC^a^ score [17]

Laboratory marker	Value	Score
C-reactive protein (mg/l)	<150	0
	≥150	4
Leukocyte count (×10^9^/l)	<15	0
	15–25	1
	>25	2
Hemoglobin (g/l)	>135	0
	110–135	1
	<110	2
Serum sodium (mmol/l)	≥135	0
	<135	2
Serum creatinine (μmol/l)	≤141	0
	>141	2
Serum glucose (mmol/l)	≤10	0
	>10	1

^a^LRINEC, Laboratory Risk Indicator for Necrotizing Fasciitis

**Table 3:** LRINEC^a^ risk stratification [17]

Score	Risk level	Probability of diagnosing NF^b^
≤5	Low	<50
6–7	Medium	50–75
≥8	High	>75

^a^LRINEC, Laboratory Risk Indicator for Necrotizing Fasciitis. ^b^NF, necrotizing fasciitis

**Table 4:** SIARI^a^ score [25]

Variable	Score
Site of infection outside of lower limb	3
History of immunosuppression	3
Age ≤ 60 years	2
Creatinine > 141 μmol/l	1
White cell count > 25 × 10^9^/l	1
C-reactive protein ≥ 150 mg/l	1

^a^SIARI, Site other than the lower limb, Immunosuppression, Age, Renal impairment, and Inflammatory markers

**Table 5:** LARINF^a^ score [26]

Variable	Value	Score
Hemoglobin (g/l)	>135	0
	110–135	1
	<110	2
Procalcitonin (ng/ml)	<1	0
	≥1	3
C-reactive protein (mg/dl)	<10	0
	≥10	1
Heart, liver, or renal insufficiency	No	0
	Yes	2
Immunosuppression	No	0
	Yes	2
Obesity	No	0
	Yes	1

^a^LARINF, Laboratory and Anamnestic Risk Indicator for Necrotizing Fasciitis

SIARI score: Cribb *et al.* [25] developed a simplified Site other than the lower limb, Immunosuppression, Age, Renal impairment, and Inflammatory markers score (Table 4) to assist in the diagnosis of NF. Unlike traditional scoring systems, the SIARI score requires fewer laboratory parameters and incorporates key clinical factors, thereby improving diagnostic accuracy. When a diagnostic threshold of 3 points is applied, the SIARI score achieves a sensitivity of 84% and a specificity of 70%. Thus, the SIARI score has a strong capacity to identify NF, thereby effectively reducing the risk of missed and misdiagnosed cases.

LARINF score: Breidung *et al.* [26] developed a novel diagnostic scoring tool for NF—the Laboratory and Anamnestic Risk Indicator for Necrotizing Fasciitis (Table 5). This scoring system integrates three laboratory indicators (hemoglobin, procalcitonin [PCT], and C-reactive protein) and three significant comorbidities (cardiac, hepatic, or renal insufficiency combined as a single parameter, immunosuppression, and obesity). The score is calculated by summing the points assigned to these six indicators. When a diagnostic threshold of 5 points is applied, the LARINF score has a sensitivity of 84% and a specificity of 75% for NF. Thus, the LARINF score is highly accurate in diagnosing NF and can serve as a valuable reference for clinical diagnosis.

Recommendation 3 (highly recommended): It is suggested that CT scans be the first choice for emergency screening, that point-of-care ultrasound be used for rapid diagnosis, and that MRI be utilized as an auxiliary diagnostic tool during stable phases. Surgical exploration and pathological examination are the gold standards for confirmation, while multiple microbiological cultures are used to identify pathogens. The use of X-ray is not recommended (evidence level: high).

Rationale: Imaging Examination: In the early stages of NF, X-ray findings are often nonspecific and similar to those of cellulitis, which is characterized primarily by soft tissue blurring and thickening. Although the presence of gas in the fascial plane is a hallmark of NF, only a subset of patients exhibit gas within soft tissues. This limits the diagnostic value of X-ray, and its use as a standalone diagnostic tool is not recommended [27]. In contrast, CT scans are more informative, revealing subcutaneous fat attenuation, fascial thickening, and small air bubbles. These features make CT the preferred imaging modality for assisting in the diagnosis of NF in the emergency setting [28, 29]. Ultrasound is a rapid and convenient imaging method that allows visualization of subcutaneous thickening, gas accumulation, fascial effusion, and even a cobblestone-like appearance of the affected area. When ultrasound detects fluid accumulation exceeding 2 mm in depth, the diagnostic accuracy for NF can reach 72.7% [28, 30–33]. MRI is considered the gold standard for diagnosing soft tissue infections, with a sensitivity of 90%–100% and a specificity of 50%–85% for NF. MRI provides clear visualization of deep fascial thickening, effusion, and involvement of multiple compartments. MRI outperforms CT in imaging deep fascial effusion and is an effective tool for differentiating NF [23, 28, 34].

Surgical exploration and histopathological examination of necrotic tissue: This is the gold standard for the diagnosis of NF. Intraoperative findings such as fascial swelling, dark gray discoloration, extensive necrosis, and typical separation between the skin, fascia, and deep muscles, along with the ease of blunt dissection of muscle and fascia, in conjunction with other preoperative and intraoperative findings, strongly suggest the presence of NF. During debridement, fascial tissue is obtained for rapid frozen section biopsy. Pathological changes, including tissue necrosis and inflammatory cell infiltration, are assessed through Gram staining and culture examination, which are crucial steps in confirming the diagnosis of NF [35, 36].

Microbial detection: Repeated microbial cultures of wound exudates, affected tissues, or blood can clarify the types of pathogens and their antibiotic sensitivity profiles, providing a basis for the rational use of antimicrobial agents. Microbial detection helps to rapidly control local soft tissue infections and systemic infections, thereby reducing the amputation rate and mortality rate of patients and improving prognosis and clinical outcomes. Currently, microbial culture and identification of specimens obtained during surgery remain the main methods for determining pathogens [37, 38].

Next-generation sequencing (NGS): Qu *et al.* employed 16S rDNA sequencing technology to identify the bacterial species responsible for infections in patients with NF. Compared with traditional microbial culture, 16S rDNA sequencing can be used to rapidly and accurately determine the bacterial taxa that cause NF. This technique provides a basis for the targeted selection of antimicrobial agents and shows promise as a novel approach for rapid early diagnosis of NF. However, further research and evaluation are needed to fully establish the clinical utility of NGS [39, 40].

### Clinical question 3: general treatment of necrotizing fasciitis

In the management of NF, early diagnosis and timely, appropriate treatment are crucial for successful outcomes. These strategies primarily encompass the following five key aspects: early diagnosis coupled with thorough debridement, broad-spectrum antimicrobial therapy, aggressive fluid resuscitation, repeated assessment of the patient's condition, and comprehensive nutritional support [41].

Recommendation 4 (highly recommended): In cases where NF is highly suspected, surgical exploration should be conducted without delay. Concurrently, active measures must be taken to ensure stability of respiration, circulation, and overall homeostasis (evidence level: moderate).

Rationale: Before initial debridement and without causing any delay, the patient’s overall condition should be optimized to the greatest extent possible, which involves the following steps: maintaining electrolyte balance; correcting for hypoalbuminemia and anemia; stabilizing blood glucose levels in diabetic patients; aggressively performing fluid resuscitation for sepsis patients, with the use of inotropic or vasopressor agents as needed; providing various critical care measures, such as arterial catheterization, venous catheterization, indwelling urinary catheters, nasogastric tube placement, endotracheal intubation, and mechanical ventilation, while taking care to prevent catheter-related infections; considering hemopurification therapy for patients with acute kidney injury after identifying the underlying cause; providing intravenous nutritional support and managing with enemas and fasting for infections involving special sites, such as the perineum or perianal area; and administering targeted supplementation of coagulation factors, platelets, or other blood products for patients with coagulopathy, as well as administering antifibrinolytic agents if necessary [8, 42, 43].

### Clinical question 4: antimicrobial therapy for necrotizing fasciitis

Recommendation 5 (highly recommended): In the anti-infective treatment of NF, when the causative pathogens are not yet identified, early empirical administration of sufficient and appropriate broad-spectrum antimicrobial agents is recommended. This should be guided by epidemiological data and rapid Gram stain results to ensure coverage of both aerobic and anaerobic bacteria. The antimicrobial regimen should be promptly adjusted on the basis of pathogen identification and dynamic changes in infection markers (evidence level: high).

Rationale. In the anti-infective treatment of NF, when the pathogen has not yet been identified, systemic antimicrobial therapy is essential to reduce the bacterial load and inhibit toxin production. The following empirical antimicrobial regimens are recommended: (i) clindamycin + meropenem + vancomycin, (ii) piperacillin/tazobactam + vancomycin + clindamycin, (iii) imipenem/cilastatin + vancomycin, and (iv) ceftriaxone + clindamycin + vancomycin. During treatment, antimicrobial therapy should be promptly adjusted on the basis of bacterial culture, genetic testing, and antimicrobial susceptibility results to ensure the use of effective agents. Specimens of necrotic tissue and exudates should be collected for microbiological culture during each surgical debridement to guide subsequent antimicrobial therapy. Given the rarity of NF and the variability in microbiological features and disease severity, there is no standardized duration for antimicrobial therapy. Generally, initial antimicrobial treatment should be maintained for at least 48–72 hours; for patients with severe disease, the duration may be extended to 2 weeks. If a patient has septic shock or severe organ dysfunction, treatment should follow the bundle therapy protocol in the Sepsis-3 guidelines. This includes the selection of highly effective, broad-spectrum, and rapidly acting antimicrobial agents, along with rapid fluid resuscitation and the use of vasopressors to maintain tissue perfusion and oxygenation [44–48].

### Clinical question 5: surgical treatment of necrotizing fasciitis

Recommendation 6 (highly recommended): Incision, debridement, and drainage are essential components of emergency treatment for NF. Surgery should be performed promptly when fluctuance or significant necrotic areas are detected on the skin. During the procedure, methylene blue can be used to mark necrotic tissues, and multiple samples should be obtained for microbiological analysis. The initial surgery should be swift, straightforward, and thorough, with an emphasis on opening subcutaneous cavities and avoiding excessive debridement that may complicate subsequent wound repair. Subsequent debridement should be repeated until all the necrotic tissues are completely removed (evidence level: high).

Rationale: Debridement timing: Incision and debridement surgery is the most critical component in the treatment of NF. Early and aggressive surgical debridement can effectively halt the spread of infection and prevent systemic complications caused by the release of inflammatory mediators. Delayed surgical intervention is closely associated with high mortality rates. Studies have shown that debridement delayed beyond 24 hours after hospital admission is an independent risk factor for increased mortality, with the relative risk of death increasing by nine-fold [48–50]. After the initial debridement, if the patient’s condition permits, transfer to a tertiary general hospital with multidisciplinary collaborative treatment capabilities is recommended. These hospitals have greater expertise in managing complex and severe wounds associated with NF, as well as in subsequent tissue reconstruction and rehabilitation therapies [15, 45, 50–55].

Debridement technique: During debridement, incisions should be initiated at the skin with obvious necrosis or at the center of the lesion. The incision must traverse the infected area and progressively extend outward until healthy soft tissue is encountered, as indicated by the presence of bleeding. If the skin opening is small and the wound contains underlying cavities or sinus tracts, 2–4 ml of methylene blue can be injected into the sinus tract through the opening [56], which will stain the necrotic tissue blue, thereby facilitating more thorough debridement. During the procedure, the use of diluted epinephrine injections should be avoided; although they may reduce bleeding, epinephrine injections can also lead to the spread of infection along fascial planes or compromise tissue viability. Hemostasis can be achieved using electrocautery if bleeding occurs. During debridement, repeated irrigation of the wound with a broad-spectrum antimicrobial solution (e.g. 0.025% sodium hypochlorite or hydrogen peroxide) followed by thorough rinsing with normal saline is recommended. However, for wounds with small drainage openings and large underlying cavities, hydrogen peroxide irrigation is not advised to prevent the risk of gas embolism [8, 15, 52].

Assessment of debridement effectiveness: During surgery, multiple samples should be collected from various sites (e.g. the wound bed, wound edges, and surrounding tissues) for microbiological and histological evaluation, which to accurately identify the causative pathogens, thereby guiding the selection of appropriate antimicrobial agents and antimicrobial dressings. Pathological examination of the wound edge tissues after debridement can also confirm whether the debridement has been thorough. Following effective debridement, the patient's clinical indicators of infection, such as body temperature, white blood cell count, neutrophil percentage, and PCT, should gradually normalize. Local symptoms, including pain and swelling, should be alleviated, and the patient's overall condition should significantly improve. Additionally, imaging methods, such as ultrasound, CT, and MRI, can be used to monitor the internal healing of the wound and to determine whether any sinus tracts or underlying cavities remain [2, 8, 15, 46, 52, 57].

Recommendation 7 (weakly recommended): Following thorough debridement, negative pressure wound therapy (NPWT) may be utilized to facilitate wound healing. However, NPWT should be applied cautiously in cases of confirmed anaerobic infection. In such instances, the use of antimicrobial dressings for wound care is recommended as an alternative (evidence level: moderate).

Rationale: Negative pressure wound therapy: NPWT facilitates wound healing by reducing bacterial invasion and colonization, inhibiting bacterial proliferation, minimizing the accumulation of inflammatory mediators and lactate, enhancing microcirculation, and eliminating dead space. These actions collectively create an optimal microenvironment for wound healing. Additionally, NPWT provides active drainage, and when NPWT is combined with irrigation, it can effectively remove necrotic tissue and exudates, promote granulation tissue growth, and accelerate the healing process. The use of NPWT is appropriate when the following conditions are met: (i) the wound has been thoroughly debrided of necrotic tissue, and infection is controlled, including in concealed areas such as fascial compartments and tissue spaces, and (ii) the wound has a low risk of bleeding, and the patient does not have severe coagulopathy [46, 58–60].

Antimicrobial dressings: Wound dressing is a fundamental approach in the management of NF. Postoperative wounds in NF often exhibit significant exudate. Therefore, highly absorbent dressings such as silver ion foam dressings, silver ion alginate dressings, or sulfadiazine silver hydrocolloid dressings are recommended. These dressings help prevent exudate from macerating the surrounding skin while effectively draining necrotic tissue and reducing the frequency of dressing changes. For wounds with substantial residual necrotic tissue, silver-containing dressings can be used for debridement. To alleviate pain during dressing changes, nonadhesive dressings such as hydrophilic fiber dressings or hydrogel dressings are preferred. In cases where debridement results in a noticeable cavity, foam dressings can be used to fill the space and should be changed frequently. Research has demonstrated that effectively managing wound exudate and maintaining a moist wound environment promote epithelial cell migration and create optimal conditions for granulation tissue growth and wound epithelialization [61–63].

Recommendation 8 (highly recommended): Amputation should be considered with caution. The main indications include uncontrollable and life-threatening limb infections or when limb function is beyond salvage. However, the decision and timing of surgery should be based on a comprehensive assessment of the patient's overall clinical status (evidence level: moderate).

Rationale: Although the overall amputation rate in NF patients is relatively low, significant disparities exist across different socioeconomic groups. Specifically, patients from low-income backgrounds have a higher amputation rate than those from high-income groups (*P* < .05) [64]. According to the literature, the reported amputation rate among NF patients is 8.4%, with the most common sites being the fingers/toes (44.1%) and above the knee (22.8%). Clinical predictors of amputation in NF patients include DM, soft tissue swelling, skin necrosis, gangrene, and serum creatinine ≥ 1.6 mg/dl at admission. Early initiation of broad-spectrum antimicrobial therapy and surgical debridement, combined with effective glycemic control during the perioperative period, can significantly reduce the amputation rate [43]. Amputation should be reserved for limbs with extensive muscle necrosis due to secondary infection where function cannot be restored. If the condition does not improve after debridement and the fasciitis continues to spread extensively, posing a life-threatening risk, a secondary amputation may be necessary [65]. Studies have shown that NF patients with hemorrhagic bullae, peripheral vascular disease, LRINEC scores > 8, or bacteremia have significantly higher mortality rates with late amputation than with early amputation [66]. Additionally, case reports have documented limb salvage through amputation in patients with lower limb necrosis due to spreading infection [67]. Another study has revealed that during and after the COVID-19 pandemic, the incidence of NF significantly increased and was associated mainly with infections caused by *S. pyogenes*; these cases exhibited high invasiveness, resulting in amputation or death in more than half of the patients [68].

Recommendation 9 (highly recommended): When treating Fournier's gangrene (FG), it is crucial to evaluate underlying anorectal conditions, such as anal fistulas. During surgical debridement, meticulous care should be taken to preserve the testes unless severe infection or clear necrosis is present. Injury to the rectum, anal canal, and anal sphincter must be avoided. Strict perioperative management of bowel and bladder function is essential to prevent wound contamination. Fasting and parenteral nutrition support may be indicated if necessary (evidence level: moderate).

Rationale: FG is an acute necrotizing infection that primarily affects the scrotum, perineum, and perianal regions. Recent studies have indicated that the use of sodium–glucose cotransporter-2 inhibitors for treating diabetes, chronic kidney disease, and cardiovascular conditions may increase the risk of developing FG [69–71]. In the management of FG in male patients, orchiectomy is typically unnecessary because of the distinct vascular supply of the testes and scrotum [8, 72]. However, in cases of severe testicular infection or necrosis, orchiectomy may be considered after a thorough assessment by a urologist. Research has shown that ~21% of patients require unilateral orchiectomy. The presence of intratesticular gas detected by imaging suggests the presence of nonviable testicular tissue, warranting orchiectomy and potentially reducing mortality by 70% [73]. To prevent fecal and urinary contamination of the surgical wound, a urinary catheter and fecal diversion bag should be placed preoperatively. However, iatrogenic urethral injury and worsening of FG are potential complications of catheterization; therefore, forceful catheter insertion should be avoided if resistance is encountered [74]. Some researchers advocate suprapubic cystostomy as an effective means of urinary management, facilitating subsequent urethral interventions [69]. Additionally, colostomy has been shown to reduce wound infection rates and accelerate wound healing [75–78]. Preoperative consultation with a general surgeon is essential to assess the need for fecal diversion and to discuss several details, such as stoma site, method of creation (laparoscopic vs open surgery), and potential timing for stoma reversal [77]. In cases where fecal contamination of the wound is highly likely and no diversion is performed, fasting is recommended to minimize risk.

Recommendation 10 (weakly recommended): Wound reconstruction is a core therapeutic goal in the management of NF, and it should be performed once the local infection is controlled, the wound bed is adequately prepared, and the patient’s overall condition is stable. The choice of reconstruction method depends on the wound characteristics and may include direct closure, skin grafting, or flap transfer. For complex wounds with exposed bone or tendons or those involving sinus tracts, reconstruction can be enhanced with the use of certain materials, such as antibiotic-loaded cement spacers, platelet-rich plasma (PRP), or artificial dermal scaffolds (evidence level: moderate).

Rationale: For patients with small wound defects and low local tissue tension, direct closure is typically appropriate. If the wound bed is clean and the granulation tissue is robust, split-thickness skin grafting can be performed directly. Most NF patients are well suited for free skin grafting to repair their wounds. For nonfunctional areas, thin split-thickness grafts (0.2–0.25 mm) are recommended, whereas medium-thickness grafts (0.35–0.8 mm) are preferred for functional areas. In complex wounds, such as those with exposed deep tissues or compromised vasculature and nerves, a combination of skin grafting and flap transfer can be employed to achieve optimal repair outcomes. The survival rate of both skin grafts and flaps is closely related to the quality of the granulation tissue and the underlying blood supply. Therefore, dynamic monitoring of the blood supply to transplanted grafts or flaps is essential to ensure the safety and efficacy of the wound repair process [78, 79].

Antibiotic-loaded bone cement: Antibiotic-loaded bone cement is composed of polymethylmethacrylate (PMMA) combined with antibiotics, and it is used for local treatment of NF. Antibiotic-loaded bone cement works by slowly and continuously releasing antibiotics directly to the site of infection, maintaining high local drug concentrations to effectively control the infection. This approach reduces the need for systemic antibiotics, thereby minimizing adverse systemic effects. Antibiotic-loaded bone cement has the following key advantages: (i) high local drug concentration, because directly acting on the infected site bypasses the limitations of poor local blood supply, which often prevents systemic antibiotics from reaching the infection effectively, thus ensuring more efficient eradication of pathogens; (ii) extended duration of action, owing to its controlled-release mechanism, which allows the drug to remain active locally over an extended period, thereby reducing the frequency of dressing changes and minimizing the risk of recurrent infection; (iii) dead space filling, as this method can fully contact the wound and fill residual cavities after debridement, preventing hematoma and fluid accumulation, which reduces bacterial residue and lowers the risk of infection recurrence; and (iv) a high safety profile, owing to the drug acting locally with minimal systemic absorption, resulting in low systemic toxicity. The commonly used antibiotics include broad-spectrum agents such as vancomycin, gentamicin, and tobramycin. After application, an induced membrane, which is rich in bone morphogenetic proteins and vascular endothelial growth factors, forms over the wound, which promotes neovascularization, accelerates wound healing, and enhances resistance to infection. The formation of the induced membrane also serves as a marker of thorough debridement. In the second-stage surgery, after removal of the antibiotic-loaded bone cement, direct skin grafting can be performed over the induced membrane. Studies have shown that patients treated with antibiotic-loaded bone cement require an average of three surgeries, which is significantly fewer than the number of surgeries required by those treated with traditional “nibbling” debridement. Additionally, limb salvage rates are markedly improved using this method, with no reported fatalities [80–87].

Platelet-rich plasma: PRP is an emerging biologic therapy enriched with leukocytes, cytokines, growth factors, and chemokines. These bioactive components modulate inflammation and promote tissue repair and regeneration. Research has indicated that using PRP with a high concentration of leukocytes and minimal red blood cells to fill wounds significantly increases blood flow, stimulates granulation tissue growth, and expedites wound healing, yielding favorable clinical results [88, 89].

Artificial dermal scaffold: An artificial dermal scaffold (ADS) is a bilayered biomaterial used for skin repair, and it is designed to replicate the structure and function of normal human skin. An ADS promotes cell migration and angiogenesis, supporting the regeneration and permanent reconstruction of dermal tissue. Moreover, an ADS directly covers exposed bone and tendons, facilitating rapid vascularization and preventing tendon adhesion. Thus, an ADS is an optimal approach for wound bed preparation [90–92].

### Clinical question 6: adjuvant therapy for necrotizing fasciitis

Recommendation 11 (highly recommended): For patients with impaired consciousness, respiratory failure, or NF involving the face and neck, close monitoring of local edema and respiratory status is essential. When necessary, a prophylactic artificial airway should be established. Respiratory support measures, including oxygen therapy, assisted ventilation, or mechanical ventilation, should be applied in a stepwise manner on the basis of oxygenation and pulmonary status (evidence level: low).

Rationale: Airway management is critical for patients with NF of the neck, as this disease can cause cervical edema and tissue necrosis, significantly increasing the difficulty of tracheal intubation. Studies have demonstrated that for patients with mild (Grade 1) or moderate (Grade 2) respiratory distress, short-term corticosteroid therapy (e.g. 10 mg of dexamethasone) combined with early surgical debridement and drainage can often avoid the need for tracheostomy. However, for those with severe (Grade 3) respiratory distress, tracheostomy should be performed immediately if corticosteroid therapy is ineffective. In cases where patients have concurrent pulmonary infections and poor oxygenation, a stepwise respiratory support strategy, including oxygen therapy, assisted ventilation, or mechanical ventilation, may be considered. For patients with neck NF requiring long-term airway management, tracheostomy is generally preferred over orotracheal or nasotracheal intubation because tracheostomy is safer, more secure, and reduces the risk of complications [93–95].

Recommendation 12 (highly recommended): High-dose corticosteroids are not recommended. Intravenous immunoglobulin (IVIG) may be considered for immune supportive therapy; however, its potential risk of renal injury should be carefully monitored (evidence level: moderate).

Rationale: IVIG is a therapeutic modality with anti-inflammatory and immunomodulatory properties. IVIG neutralizes bacterial exotoxins and antigens and inhibits the activity of virulence factors produced by group A streptococcus (GAS), thereby reducing disease severity. Studies have demonstrated that IVIG can be used as an adjunctive therapy in patients with NF of the neck, facilitating recovery [96]. The potential mechanisms of IVIG involve inhibiting superantigen activity, reversing excessive T-cell proliferation, and downregulating tumor necrosis factor (TNF), which block the cytokine cascade reaction mediated by superantigens. In patients with severe GAS infections, IVIG has been shown to reduce mortality. However, Kadri *et al.* indicated that IVIG has no significant effect on mortality or hospital stay duration in patients with NF. Therefore, some experts have suggested that IVIG should be reserved for critically ill patients with necrotizing soft tissue infections caused by *Staphylococcus* or *Streptococcus* [97–99]. Additionally, while some scholars have proposed early and short-term use of high-dose corticosteroids as an adjunct to adequate and effective antimicrobial therapy [100], Petitpas *et al.* [101] revealed that early corticosteroid use may mask natural disease progression and clinical signs, potentially affecting diagnostic accuracy and therapeutic decision-making.

Recommendation 13 (highly recommended): Following confirmation of the etiology, a tiered pain management strategy is recommended. Patients with NF are at high risk for deep vein thrombosis (DVT); therefore, a tiered DVT prophylaxis strategy is advised (evidence level: low).

Rationale: Stratified/Multimodal analgesia: Patients with NF often experience severe pain during treatment, which can significantly impact both physical and psychological recovery, as well as increasing the risk of developing abnormal pain or allodynia. Nonspecific pain is an important clinical manifestation of NF progression; therefore, the location, intensity, and nature of pain should be closely monitored early in the disease course. If pain worsens, the possibility of disease progression should be ruled out first to avoid the indiscriminate use of sedative or analgesic medications. Once the cause of pain is identified, nonsteroidal anti-inflammatory drugs (NSAIDs), such as parecoxib or flurbiprofen ester, are recommended. For patients with extensive surgical procedures or significant postoperative pain, a multimodal analgesia strategy may be employed, such as the use of hibernation agents, intramuscular injection of pethidine, or oral administration of tramadol hydrochloride. Importantly, NSAIDs may affect the coagulation function and the gastric mucosa, whereas centrally acting analgesic and sedative drugs may suppress respiratory and circulatory functions. For stratified management of mild, moderate, and severe pain, the Expert Consensus on Emergency Trauma Pain Management is recommended. Analgesics of different levels and mechanisms should be selected on the basis of pain assessment results to optimize pain control [15, 45, 102–104].

Stratified management of DVT: Patients with NF are at high risk for DVT due to prolonged illness and extended bed rest, which can lead to sluggish blood flow. DVT not only impairs local recovery but also exacerbates underlying conditions and worsens overall clinical status. Therefore, upon admission, patients should receive education on thromboprophylaxis, with the affected limb elevated and encouraged to engage in active and passive movement exercises. A venous thromboembolism risk assessment should be conducted within 24 hours of admission and immediately after each surgical procedure. If necessary, plasma D-dimer levels should be measured and diagnostic imaging, such as color Doppler ultrasound, CT venography, or MRI venography, should be performed. On the basis of the assessment and diagnostic results, therapeutic measures, such as anticoagulation, thrombolysis, inferior vena cava filter placement, or surgical thrombectomy, may be considered. For specific protocols, reference can be made to the Guidelines for the Diagnosis and Treatment of Deep Vein Thrombosis (Third Edition) [105, 106].

Recommendation 14 (weakly recommended): For patients with NF who have stable systemic conditions and can be safely transported, hyperbaric oxygen therapy (HBOT) is recommended (evidence level: high).

Rationale. HBOT serves as an effective adjunctive treatment for NF. By increasing tissue oxygen levels, HBOT reduces the release of exotoxins (such as lecithinase) and effectively inhibits the growth of anaerobic bacteria, thereby alleviating the severity of infection. Additionally, HBOT decreases leukocyte chemotaxis and adhesion, mitigates ischemia-reperfusion injury, suppresses the formation of inflammatory mediators, promotes angiogenesis, enhances tissue regeneration, and accelerates the healing process [107–110]. Both domestic and international studies have demonstrated the successful use of HBOT in treating perianal NF. These findings indicate that HBOT can improve local tissue oxygenation, creating favorable conditions for wound healing and significantly improving patient outcomes. In patients with concurrent septic shock, HBOT can reduce inflammatory responses, control sepsis, and increase survival rates [111]. Furthermore, research has indicated that HBOT can lower mortality rates, lower amputation rates, shorten hospital stays, and reduce the number of surgical debridements in patients with NF [112, 113].

Recommendation 15 (highly recommended): Early initiation and continuous implementation of individualized comprehensive rehabilitation and psychological therapy are recommended throughout the entire treatment process. Nutritional support should also be integrated across the full treatment cycle (evidence level: moderate).

Rationale: Rehabilitation and psychological therapy: When conducting a comprehensive rehabilitation assessment for patients with NF, it is crucial to consider multiple factors, including the patient’s age, the location of the lesion, underlying comorbidities, premorbid activities of daily living (ADLs), stages of surgical intervention, and severity of NF. Additionally, the recovery of postoperative ADLs and the degree of scar hyperplasia should be integrated to develop a tailored rehabilitation plan. The rehabilitation plan should encompass various aspects, such as active and passive movement training, physical modality therapy, orthotic immobilization, scar management, and functional rehabilitation exercises [45]. The treatment course for NF is often lengthy, and patients frequently require multiple surgeries, leading to significant medical expenses. Moreover, NF predominantly affects the lower limbs, causing extensive lesions that result in prolonged bed rest and restricted mobility, which is particularly true for patients who have undergone amputation, where functional impairment is more pronounced. During the treatment process, patients also experience severe pain, which significantly disrupts their normal rest and substantially increases their psychological burden, often leading to prolonged states of anxiety. Therefore, timely psychological counseling or therapy is highly important. Studies have shown that posttraumatic psychological stress can be effectively prevented and improved through early intervention. Medications, such as alprazolam, fluoxetine, and olanzapine, can be administered orally to alleviate anxiety and improve sleep quality [114].

Nutritional support: Nutritional support is essential for patients with NF, as extensive wounds result in significant loss of fluids and proteins. NF patients have high metabolic demands and require comprehensive nutritional assessment and risk screening. When the nutritional risk screening score exceeds 3, nutritional support therapy should be initiated. Prolonged fasting, often resulting from frequent surgeries or other interventions, may lead to malnutrition, which can impair fibroblast proliferation, disrupt neovascularization, and weaken the immune system. For patients with FG, enteral nutrition can be provided if a colostomy is in place. If no colostomy is present, parenteral nutrition is recommended to achieve fecal diversion, thereby reducing the need for colostomy and maximizing patient benefits. Studies have shown that early nutritional support is a critical factor in improving survival rates among NF patients, who may require up to twice their basal energy needs. Therefore, a detailed nutritional management protocol is recommended on the basis of the Clinical Guidelines for Parenteral and Enteral Nutrition in Adult Patients in China (2023 Edition) [8, 115–118].

## Conclusion

**Figure 1:**
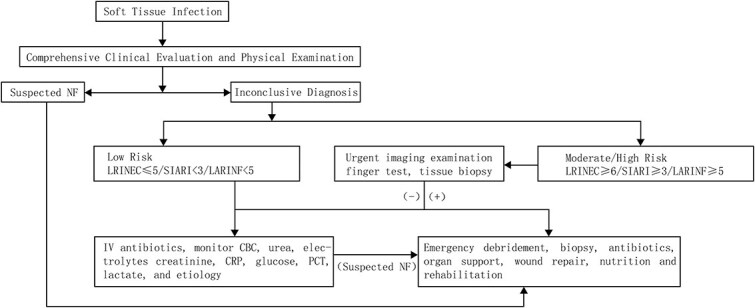
Simplified workflow for the diagnosis and management of NF.

Given the paucity of clinical evidence and the dearth of pediatric treatment experience for NF, this consensus predominantly centers on surgical management strategies. The consensus aims to synthesize prior clinical experiences in NF management and provide clinicians with a scientifically robust and standardized decision-making framework. When implementing this consensus, clinicians should meticulously evaluate the unique clinical context and characteristics of each patient’s condition, conduct a thorough assessment, and formulate individualized treatment plans accordingly. The flowchart of expert recommendations is depicted in Figure 1. Early diagnosis of NF remains fraught with challenges, and its management is impeded by limitations in disease recognition, diagnostic modalities, therapeutic interventions, and patient care protocols. To surmount these obstacles, we propose the following strategic imperatives: enhancing diagnostic acumen through advanced diagnostic techniques, developing novel antimicrobial agents targeted against causative pathogens, advocating minimally invasive surgical debridement, employing biologic agents to modulate the inflammatory response, instituting comprehensive rehabilitation protocols, engaging in multidisciplinary collaborative care, and leveraging cutting-edge technologies such as artificial intelligence for diagnostic and prognostic enhancement. These measures are anticipated to significantly augment diagnostic precision and therapeutic efficacy for NF, thereby overcoming existing therapeutic limitations.

## Notes

Editorial group for the Consensus on the Diagnosis and Treatment of Adult Necrotizing Fasciitis (2025 Edition)

**Consensus cochairs** 

Institute of Burn Research, State Key Laboratory of Trauma and Chemical Poisoning, the First Affiliated Hospital of Army Medical University (the Third Military Medical University), Gaoxing Luo

**CANF expert panel members** 

First Affiliated Hospital of Anhui Medical University, Xulin Chen; Affiliated Fuyang Hospital of Anhui Medical University, Qinglian Xu; Union Hospital Affiliated to Fujian Medical University, Zhaohong Chen; Gansu Provincial People's Hospital, Jian Wu; Guangzhou Red Cross Hospital, Xiaojian Li; Third Hospital of Hebei Medical University, Qingfu Zhang; First Affiliated Hospital of Naval Medical University, Shizhao Ji and Shichu Xiao; Heilongjiang Provincial Hospital, Rui Liu; First Hospital of Jilin University (Norman Bethune First Hospital), Jiaao Yu; Affiliated Hospital of Jiangnan University, Guozhong Lu; Southern Theater General Hospital of the People's Liberation Army, Biao Cheng; Fourth Medical Center of the General Hospital of the People's Liberation Army, Chuanan Shen; Translational Medical Research Center, Department of Medical Innovation Research, General Hospital of the People's Liberation Army, Yongming Yao; First Affiliated Hospital of Air Force Medical University, Hao Guan, Dahai Hu, and Juntao Han; Second Affiliated Hospital of Kunming Medical University, Wenjun Liu; Second Hospital of Lanzhou University, Yi Liu; First Affiliated Hospital of Army Medical University (Third Military Medical University), Fei Luo, Wenhao Shen, Bo Tang, Haisheng Li, Gaoxing Luo, Ling Zhou, Zhiqiang Yuan, and Yizhi Peng; First Affiliated Hospital of Nanchang University, Hongyan Zhang and Guanghua Guo; Affiliated Hospital of Nankai University (Tianjin Fourth Hospital), Yongjian Zhao; Affiliated Hospital of Nantong University, Yi Zhang; Ruian People's Hospital, Jiong Chen; Ruijin Hospital Affiliated to Shanghai Jiao Tong University School of Medicine, Yan Liu; Beijing Jishuitan Hospital Affiliated to Capital Medical University, Yuming Shen; Beijing Children's Hospital Affiliated to Capital Medical University, Yanni Wang; West China Hospital of Sichuan University, Junjie Chen; First Affiliated Hospital of Shenzhen University, Jun Wu and Jingning Huan; General Hospital of Taiyuan Iron and Steel (Group) Co., Ltd, Peng Duan; Wuhan Third Hospital (Tongren Hospital Affiliated to Wuhan University), Deyun Wang and Weiguo Xie; Second Affiliated Hospital of Zhejiang University School of Medicine, Chunmao Han; Zhengzhou First People's Hospital, Chengde Xia; *Chinese Journal of Burns and Wounds*, Guangping Liang; Xiangya Hospital of Central South University, Pihong Zhang and Pengfei Liang; First Affiliated Hospital of Sun Yat-sen University, Julin Xie

**Methodology expert** 

Center for Evidence-Based Nursing, West China Hospital, Sichuan University, Cong Wang

**Writing group** 

Institute of Burn Research, the First Affiliated Hospital of Army Medical University (the Third Military Medical University), Ling Zhou and Haisheng Li

**Members of the editorial team** 

Institute of Burn Research, the First Affiliated Hospital of Army Medical University (the Third Military Medical University), Dongdong Xue, Wei Zhu, Songwei Zhou, Yanjun Wu, Yali Gong, Qimeng Li, and Yu Mo

**Evidence team members** 

Institute of Burn Research, the First Affiliated Hospital of Army Medical University (the Third Military Medical University), Ning Li, Huapei Song, Siyuan Ma, Fei Xiang, Junyi Zhou, Wei Qian, Rixing Zhan, and Jianglin Tan

**Clinical problem solicitation expert group** 

Panelists of the Consensus Committee.
